# The impact of intravenous iodinated contrast agents on radiotherapy dose calculation and radiobiological effect predictions in central lung cancer

**DOI:** 10.3389/fonc.2025.1563919

**Published:** 2025-08-11

**Authors:** Defu Yang, Feng Shang, Ying Xu, Ying Yan

**Affiliations:** Department of Radiation Oncology, General Hospital of Northern Theater Command, Shenyang, China

**Keywords:** central lung cancer, radiotherapy, iodine ion contrast, AXB algorithm, AAA algorithm

## Abstract

**Background:**

Accurate dose calculation algorithms are critical for optimizing radiotherapy outcomes. This study evaluates and compares dosimetric differences and predictions of Tumor Control Probability (TCP) and Normal Tissue Complication Probability (NTCP) between the Analytic Anisotropic Algorithm (AAA) and Acuros XB (AXB) algorithm in lung cancer radiotherapy, under both contrast-enhanced and non-contrast enhanced CT conditions.

**Methods:**

Twenty patients with centralized lung cancer treated with intensity-modulated radiation therapy (IMRT) technique, including two patients with small cell lung cancer and 18 with non-small cell lung cancer, were selected to undergo CT scanning with and without contrast. Multiple dosimetric parameters were calculated for both algorithms under enhanced and non-contrast enhanced CT conditions. TCP and NTCP were assessed for critical organs such as the lungs, heart, and esophagus.

**Results:**

Significant differences were observed in dosimetric values between the AAA and AXB algorithms. For the minimum dose (PTV_Min), the AAA algorithm yielded higher values under enhanced CT conditions (4427 cGy) compared to non-contrast enhanced CT (3872 cGy), whereas AXB showed 4248 cGy (enhanced CT) and 3762 cGy (non-contrast enhanced CT). For maximum dose(PTV_Max), The AAA algorithm showed 6430 cGy (enhanced CT) compared to AXB's 6541 cGy (p< 0.0001). The mean dose (PTV_Mean) was 5674 cGy for AAA vs. 5640 cGy for AXB (p = 0.0042). TCP analysis showed that AAA predicted higher TCP values across both imaging conditions, with a 0.69% difference between AXB_C_Dm and AXB_C_Dw under enhanced CT (p = 0.0011). NTCP for lung radiofibrosis was 20.42% higher with the AAA algorithm, suggesting increased risk.

**Conclusion:**

The AAA algorithm tends to overestimate both tumor control and normal tissue complications, while the AXB algorithm provides more conservative estimates. These findings highlight the importance of algorithm choice in optimizing treatment planning and minimizing adverse effects in radiation therapy.

## 1 Introduction

Lung cancer is the most common malignancy and the leading cause of cancer-related death in China and worldwide (1). Treatment options for lung cancer typically include surgery, chemotherapy, immunotherapy, and radiotherapy. The combination of radiotherapy and immunotherapy is often employed to enhance local tumor control, reduce or eliminate distant metastases, and ultimately improve survival rates while preserving the integrity and function of organs and tissues (2–4). Radiotherapy remains one of the primary treatment modalities for lung cancer, with over 60% of patients receiving it at some point during their disease course (5, 6).

During the radiotherapy planning phase, precise delineation of the target area and organs at risk (OARs) is crucial. The intravenous injection of contrast agents can improve the clarity of the boundaries between healthy tissue and tumors. However, contrast agents composed of elements with high atomic numbers have been shown to introduce errors in dose calculations (7). Some studies have quantified the differences in Hounsfield units (HUs) between pre-and post-contrast scans, noting significant variability that directly impacts dose calculations, with mean HU increases of 20–30 HU leading to dose calculation errors ranging from 3% to 6% (8).The attenuation of high-energy X-rays, quantified by HUs, is crucial for dose calculations, which heavily depend on the electron density and mass density derived from HU values. When enhanced CT images are used for dose calculation, the increased electron density of tissues must be accounted for to avoid dose biases (9). While previous studies have demonstrated statistically significant dose differences due to contrast agents, their clinical relevance varies depending on the tumor type (10, 11). This issue is particularly pertinent in regions with complex blood supplies, such as the thorax and abdomen, where the impact of contrast agents on dose calculations needs careful consideration. The thoracic cavity, rich in blood vessels, may exhibit substantial HU value differences between pre- and post-contrast scans, particularly in peripheral organs like the heart, which is also highly vascularized.

Given these considerations, it is essential to investigate the differential effects of intravenous high-density iodinated contrast agents on dose calculations performed using the Analytic Anisotropy Algorithm (AAA) and the Acuros XB (AXB) algorithm. The AAA and the AXB algorithm represent two advanced dose calculation models employed in radiotherapy treatment planning, each distinguished by its underlying computational framework and physical modeling approach (12, 13).The AAA algorithm is based on a convolution-superposition method, which models dose deposition by considering the spread of secondary electrons and photon scatter within a heterogeneous medium. This algorithm incorporates an anisotropic analytical kernel that accounts for variations in tissue density, allowing it to efficiently estimate dose distributions with reasonable accuracy across different tissue types (14). The AAA model divides the treatment volume into small voxels, applying pre-calculated kernels to predict dose deposition based on the electron density of each voxel. The Acuros XB algorithm is grounded in the principles of linear Boltzmann transport equations (LBTE), which provide a more comprehensive physical model of radiation transport (15). AXB explicitly solves the LBTE, accounting for both primary and scattered radiation as it interacts with complex tissue geometries and varying material compositions. This approach allows AXB to model dose deposition with high accuracy, particularly in environments with significant heterogeneities, such as lung tissues or areas affected by contrast agents. The algorithm’s ability to simulate the transport of radiation through different media with detailed physics modeling makes it highly effective in predicting dose distributions in challenging clinical scenarios.However, there remains a gap in the literature specifically addressing the impact of contrast agents on radiobiological effect predictions, such as Tumor Control Probability (TCP) and Normal Tissue Complication Probability (NTCP), especially in central lung cancer. This gap underscores the need for research that not only compares these algorithms in terms of physical dose calculations but also evaluates how these differences translate into clinical outcomes.This study aims to quantify the impact of high-density iodinated contrast agents on dose calculations using both the AAA and AXB algorithms in the presence of heterogeneous media surrounding central lung cancer targets. Furthermore, to correlate physical dose differences with clinical outcomes, this study will evaluate the effect of contrast agent-induced dose discrepancies on radiobiological effect predictions, specifically Tumor Control Probability (TCP) and Normal Tissue Complication Probability (NTCP). Through comprehensive analysis, this study seeks to enhance the accuracy and efficacy of radiotherapy treatment planning for lung cancer, ultimately improving patient outcomes.

## 2 Materials and methods

### 2.1 Simulated localization of lung cancer patients

**Figure 1:**
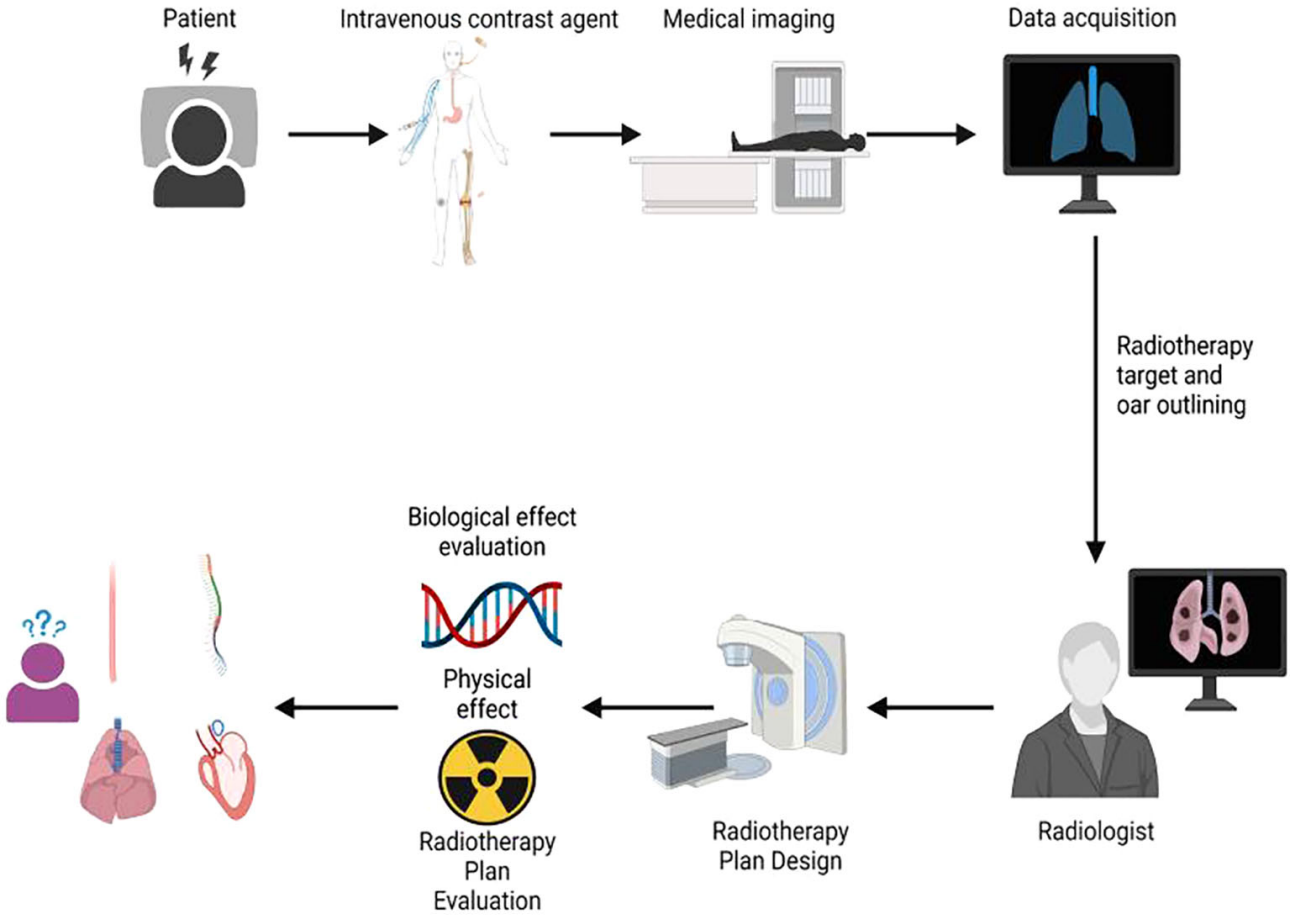
Research workflow diagram.

The workflow of this study is illustrated in **Figure 1**. All enrolled patients initially received an intravenous injection of iodinated contrast agent, followed by CT simulation, target volume delineation, radiotherapy treatment planning, and final plan evaluation. The evaluation process incorporated both physical dosimetric parameters and radiobiological modeling. A total of 20 patients with centrally located lung cancer who underwent intensity-modulated radiation therapy (IMRT) were included in this retrospective analysis. Among them, 2 patients were diagnosed with small cell lung cancer (SCLC) and 18 with non-small cell lung cancer (NSCLC). Each patient underwent two CT simulations: one with intravenous contrast and one without. Imaging was performed using a Philips Brilliance Big Bore CT scanner. Patients were positioned in the supine position, immobilized with a negative-pressure vacuum cushion, and carefully aligned to minimize setup errors due to body rotation. The sagittal laser line was used to align the midline of the body, while the mid-axillary line served as the horizontal reference. To enhance setup reproducibility, three fiducial markers were placed at the same axial level and aligned with the transverse laser plane. These markers were positioned as close as possible to the geometric center of the tumor. Before scanning, all patients received respiratory training to maintain calm and consistent breathing throughout the acquisition. The scanning range extended from the level of the cricothyroid membrane to 1 cm below the diaphragm. CT acquisition parameters were standardized as follows: tube voltage of 120 kV, tube current of 200–250 mAs, and a slice thickness of 3 mm. For the contrast-enhanced CT scans, the same imaging protocol was applied. The contrast agent Iohexol (300 mg I/mL) was administered at a dose of 80–100 mL using an injection rate of 2.0–3.0 mL/s, followed by a 50-second delay before image acquisition

### 2.2 Definition of target areas and organs at risk

**Figure 2:**
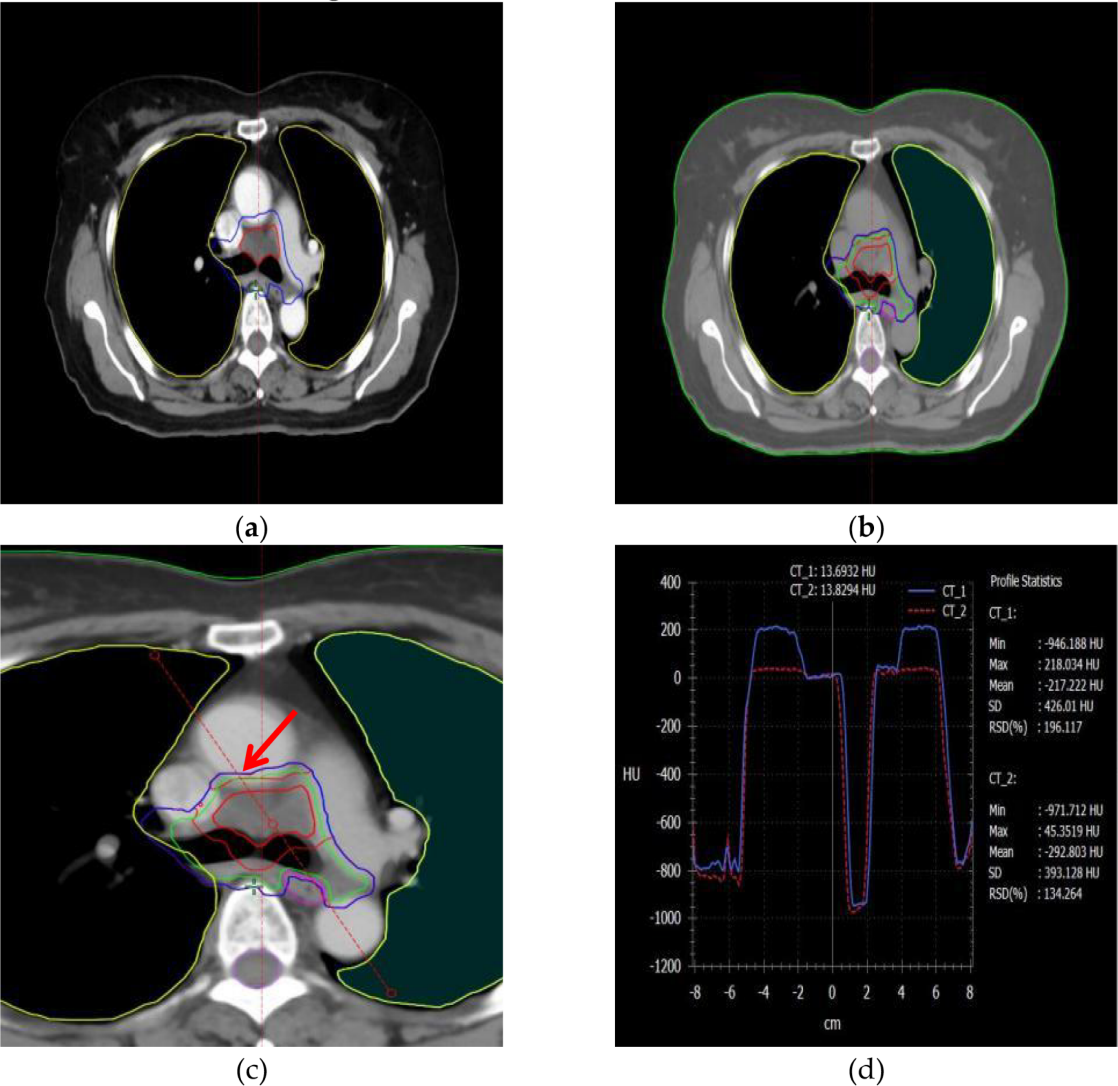
The information on the PTV and OAR structures, as well as the HU distribution curves around the target area, in plain and contrast-enhanced CT modalities. **(a)** PTV structure in the contrast-enhanced imaging modality, **(b)** PTV structure in the non-contrast (plain scan) imaging modality, **(c)** A reference line is drawn through the PTV and organs at risk (OARs) to analyze HU variations, **(d)** Changes in Hounsfield Units (HU) along the reference line under different imaging modalities.

The target volumes were delineated by radiologists with over 10 years of clinical experience. The Gross Tumor Volume (GTV) was defined as the visible intrapulmonary tumor on lung window images. The Clinical Target Volume (CTV) was created by expanding the GTV by 5–8 mm based on subclinical invasion patterns, including lymphatic regions containing involved nodes; however, prophylactic irradiation of uninvolved lymphatic drainage areas was not performed. The Planning Target Volume (PTV) was created by expanding the CTV by 5–15 mm to accommodate organ motion and setup uncertainties during treatment. The Hounsfield Unit (HU) distributions of the PTV and OAR under plain CT and contrast-enhanced CT imaging modalities are shown in **Figure 2**. Organs at risk (OARs) were contoured as follows: the spinal cord was delineated from the cricoid cartilage to the lower edge of L2 using the inner bony edge of the spinal canal; both lungs were contoured separately on lung window images, including all lung tissues regardless of expansion, collapse, fibrosis, or emphysema; the heart was contoured along the pericardium, from the inferior edge of the pulmonary trunk to the apex of the left ventricle; and the esophagus was delineated on mediastinal window images from the cricoid cartilage to the gastroesophageal junction, including the mucosal, submucosal, and muscular layers, extending outward to the surrounding adipose tissue. According to our institutional planning workflow, both non-contrast and contrast-enhanced CT images were imported into the ECLIPSE planning system. The PTV was first delineated on contrast-enhanced CT images, which were then rigidly registered to the non-contrast CT. Structures contoured on the contrast-enhanced images were transferred to the non-contrast CT and fine-tuned based on anatomical landmarks. The non-contrast CT dataset was ultimately used for treatment planning and dose calculation.

### 2.3 Radiotherapy plan design and dose calculation

For all 20 patients, IMRT planning was performed using 6 MV X-ray beams with 5–7 non-coplanar fields, evenly distributed around the target area. The beam angles were carefully selected along the longitudinal axis of the planning target volume (PTV) to minimize unnecessary irradiation of the lung, especially the healthy lung tissue, thereby reducing the low-dose lung volume. Initial plan optimization and dose calculation were conducted using the Analytic Anisotropic Algorithm (AAA). The plan quality was ensured based on ICRU62 recommendations, maintaining dose homogeneity within the PTV between +7% and -5% of the prescribed dose. The planning objectives included delivering ≥95% of the prescribed dose to ≥95% of the PTV, while avoiding excessive hotspots. Dose constraints for organs at risk (OARs) were individualized according to patient-specific prescriptions. Subsequently, the finalized AAA-based plan—including all beam parameters, structures, and monitor units (MUs)—was transferred onto the intravenous contrast-enhanced CT dataset. The dose distribution was then recalculated using the Acuros XB (AXB) algorithm without re-optimization, ensuring that the MU values and plan geometry remained unchanged. This approach allowed a direct comparison of the dose distributions between the two algorithms under identical treatment conditions.

### 2.4 PTV and OAR dose analysis

In this study, we systematically compared various dosimetric parameters for the Planning Target Volume (PTV) using contrast-enhanced and non-contrast enhanced CT images. These parameters included PTV_Min dose, PTV_Max dose, PTV_Mean dose, PTV_Median dose, PTV_Mode dose, PTV_STD, PTV_ESP, Target Area Conformity Index (PTV_CI), PTV_GM, PTV_D2% (the dose received by the highest 2% of the target area), PTV_D50%, and PTV_D98%, amounting to a total of eleven parameters for PTV evaluation. Each parameter was assessed across six data sets. To analyze the overall variability among these groups, we employed the Friedman rank-sum test, followed by Dunnett's method to examine specific differences between the dose algorithms within each group.

### 2.5 TCP and NTCP model

Based on the endpoints of the analysis, the TCP model was applied to assess the effect of contrast agent on the radiobiological effects predicted by different algorithms. Assuming that tumor control requires the killing of all tumor clonal cells, the tumor control rate (TCP) can be predicted by Poisson statistics (16) when N clonal source cell tumors are uniformly irradiated, as defined below:


$$TCP=\text{exp}\left[-Np_{s}(D)\right]$$

Cell survival is described using the single-target strike model:


$$p_{s}\;(D)=exp\;(-\alpha D)$$

Equation 1 can then be rewritten as a model with respect to two parameters (17), which are the dose and the normalized slope

${\text{γ}}_{50}$ from the phenomenological model at 50% tumor control:


$$TCP=(\frac{1}{2})\;\text{exp}\left[2\gamma_{50}(\;1-\frac{D}{D_{50}})\;/\text{ln}2\right]$$

In the case of non-uniform irradiation, if each subzone of the tumor is assumed to be independent, the tumor control rate is the product of the probability of killing all clonal cells in each subzone as depicted by the dose-volume histogram (DVH) (18):


$$TCP=\prod_{i}TCP\left(D_{i},\;v_{i}\right)$$

Thus, for a given DVH $\left(D_{i},\;v_{i}\right)$ , the TCP can be computed using the equation containing the two parameters above.


$$TCP=(\frac{1}{2})\sum_{i}v_{i}\text{exp}\left[2\gamma_{50}(1-\frac{D}{D_{50}})/\text{ln}2\right]$$

Equivalent Uniform Dose (EUD) is defined as the uniform dose of heterogeneity distributed over the entire volume of a structure, causing the same biological effect. The model was first proposed by Niemierko and the functional model is defined as follows (19):


$$EUD={\left(\sum_{i=1}({\text{v}}_{i}{\text{D}}_{i}^{\text{a}})\right)}^{\frac{1}{\text{a}}}$$

where parameter a is a parameter derived from the OAR or tumor characteristics; here parameter a for the PTV is equal to 9 (range: 8 to 10). vi Represents the partial sub-volume of the received dose Di (in Gy).

Since the prescribed dose of radiotherapy for central lung cancer is mainly limited by the dose to normal lung, esophagus, spinal cord, and heart, we focused on the normal tissue complication probability (NTCP) of these organs based on the Lyman-Kutcher-Burman (LKB) model. Lyman proposed the S-shaped dose effect (SDR), which mainly uses an integral model to estimate the dose effect of a portion of normal tissue or the whole volume after being exposed to a uniform dose of D radiation. Lyman proposed S-shaped dose response (sigmoid dose response, SDR), which mainly uses an integral model to describe the dose effect after part of the normal tissue or the entire volume is subjected to a uniform dose of D irradiation. Considering that the normal tissue is subjected to an increase in inhomogeneity of the dose, the equivalent volume method is used to improve the SDR model with the following equation (20):


$$NTCP=\frac{1}{\sqrt{2\pi }}\int_{-\infty }^{t}e^{-x^{2}/2\;}dx$$

$$t=({\text{EUD}}_{i}-{\text{TD}}_{50}(1)/\left(\text{m}\cdot {\text{TD}}_{50}\left(1\right)\right)$$

Where ${\text{TD}}_{50}\left(1\right)$ is the dose required to cause a 50% probability of complication in a given organ when the entire volume is irradiated (cGy), and m is the slope factor of the dose-response curve The LKB model calculates the probability of complication by using three parameters as well as volumetric dosimetric information, each of which has a biological effect and mathematical significance, and whose range of values is set according to clinical experience (21–26). The detailed parameters used for both the TCP and NTCP models are summarized in **Table 1**. 

**Table 1:** Lists the parameters of the TCP and NTCP model.

Structure Name	D50	m	n	α/β	End point
PTV	4920	–	–	10	TCP Poisson-LQ
Lung	2190cGy	0.80	0.37	3	Symptomatic or Radioprahic Pneumonitis(<=6months)(NTCP Lyman)
	2880cGy	0.5	0.34	3	Symptomatic or Radioprahic Fibrosis(>6months)(NTCP Lyman)
esophagus	6800cGy	–	–	3	Clinical stricture (NTCP Poisson-LQ)
	5100	0.32	0.44	10	Esophagitis, grade>=2 (NTCP Lyman)

Various evaluation indexes of the target area and organs at risk were collected and statistically analyzed by applying Graphpad Prism 9.5 software. Firstly, the overall 6 groups of data were analyzed based on Friedman rank-sum test to analyze the overall differences between the groups, and then based on Dunnett method, the specific differences between the different dose algorithms of each group were analyzed between the calculated results of the scanned CT and enhanced CT. The Dunnett-t test was used, and p less than 0.05 was considered statistically significant for differences in dose, where * p<0.05; ** p<0.01, *** p<0.001; **** p<0.0001.

## 3 Result

### 3.1 The comparison of dosimetry between the AXB algorithm and the AAA algorithm for enhanced and non-contrast enhanced CT condition

**Figure 3:**
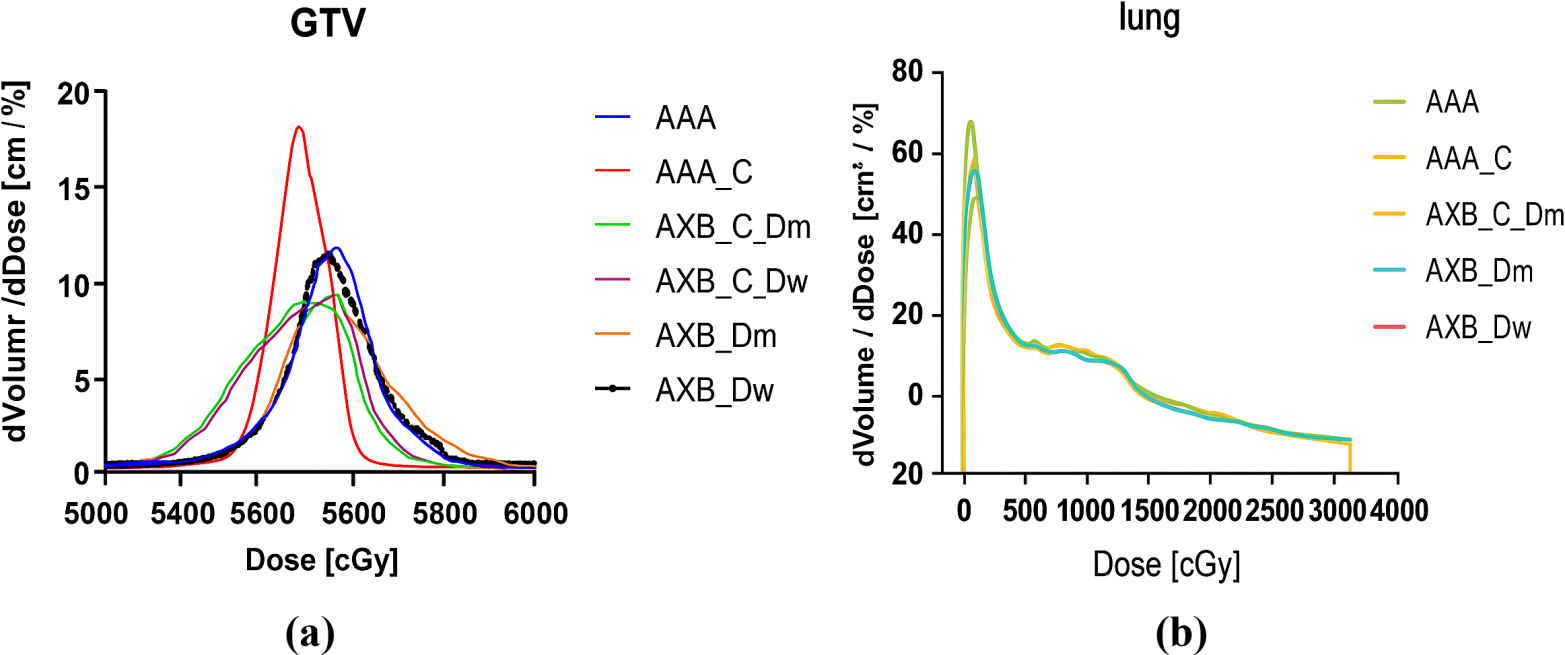
**(a)** An example of calculated dDVHs for the GTV, **(b)** An example of calculated dDVHs for the Lung.

One of the primary objectives of the Intensity-Modulated Radiation Therapy (IMRT) plan is to ensure that more than 95% of the target volume receives the prescribed dose. **Figures 3a, b** illustrate the differential Dose-Volume Histogram (dDVH) for the target area and organs at risk in a specific patient. A notable disparity is observed in the differential DVH of the Gross Tumor Volume (GTV) when comparing the Analytic Anisotropic Algorithm (AAA) to the Acuros XB (AXB) algorithm. Specifically, the dose calculations performed using the AAA on contrast-enhanced CT images are significantly higher than those obtained with the AXB algorithm. Additionally, a substantial difference is evident in the low-dose regions of the lungs in the presence of a contrast agent.

**Figure 4:**
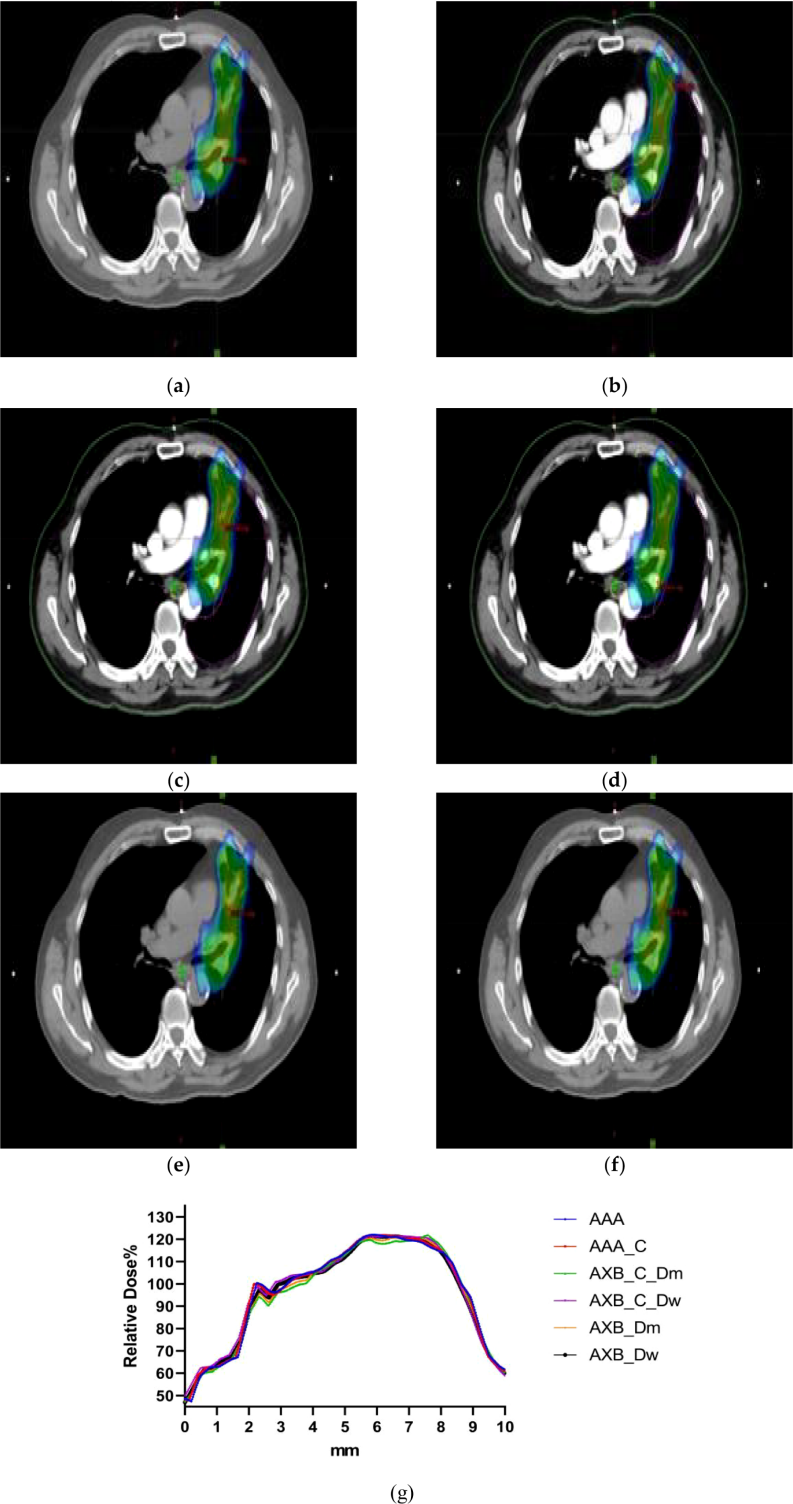
**(a–f)**The dose distribution maps of the AAA algorithm and AXB algorithm in the Dw and Dm dose reporting modes under contrast-enhanced CT and non-contrast enhanced CT conditions. **(g)** Characteristic dose curves around the tumor of a patient under different calculation conditions.

**Figure 4** illustrates the spatial dose distribution of the radiotherapy plan using both enhanced and non-contrast enhanced CT images. The dose cloud represents the spatial distribution of 100% of the prescribed dose. From the figure, it is evident that the target coverage and conformality achieved with the two different imaging modalities and algorithms meet the clinical requirements.

### 3.2 Comparison of target area dosimetry

**Figure 5:**
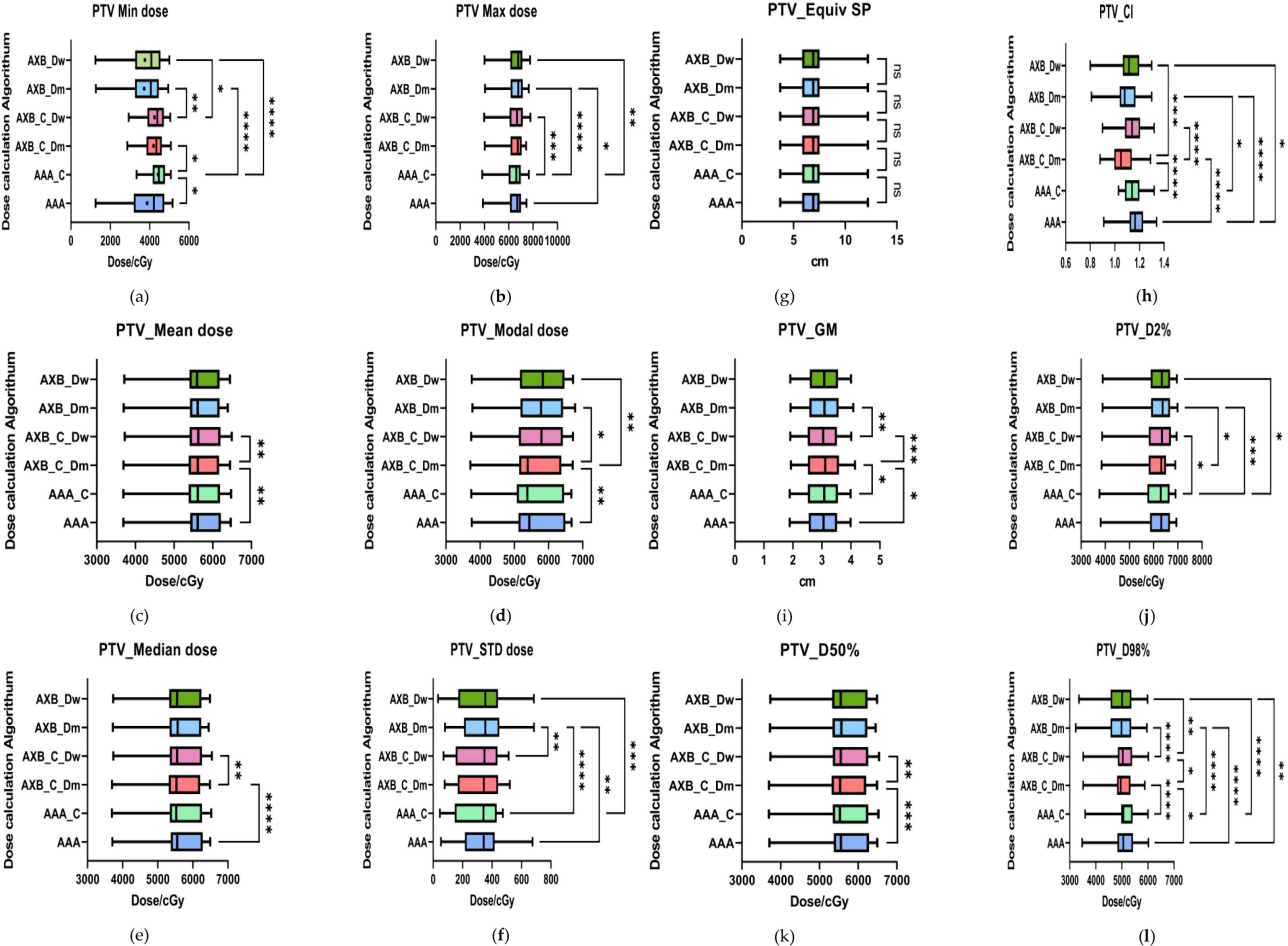
Results of target area evaluation parameters under different conditions. **(a–i)** respectively represent the comparison of PTV-related dosimetric evaluation parameters, including:PTV_Min dose, PTV_Max dose, PTV_Mean dose, PTV_Median dose, PTV_Mode dose, PTV_STD, PTV_ESP, PTV_CI, PTV_GM, PTV_D2%, PTV_D50%, and PTV_D98%. where * indicates p<0.05, ** indicates p<0.01, *** indicates p<0.001, and **** indicates p<0.0001. The abbreviation “ns” stands for “not significant,” meaning p≥0.05.

The results presented in **Figure 5** demonstrate statistically significant differences in the minimum dose (PTV_Min) across the six data sets for both the AAA and AXB algorithms (p< 0.05). Specifically, the AAA algorithm yielded higher minimum dose values under enhanced CT conditions compared to plain-scan conditions, with mean values of 4427 cGy and 3872 cGy, respectively. In contrast, the AXB algorithm, in both dose-to-water (Dw) and dose-to-medium (Dm) reporting modes, also showed higher minimum dose values under enhanced CT conditions, with mean values of 4248 cGy and 3762 cGy (Dw mode), and 4197 cGy and 3723 cGy (Dm mode), respectively. Regarding the maximum dose (PTV_Max), significant differences were observed between the AAA and AXB algorithms, particularly between the AAA_C and AXB_Dm groups, where the mean maximum doses were 6430 cGy and 6541 cGy, respectively (p< 0.0001). Similarly, the mean dose (PTV_Mean) and median dose (PTV_Median) also exhibited significant differences between the AAA and AXB algorithms. For instance, in the AAA vs. AXB_C_Dm comparison, the mean dose values were 5674 cGy and 5640 cGy, respectively (p = 0.0042).The standard deviation of the dose within the PTV (PTV_STD) and the Target Area Conformity Index (PTV_CI) showed that the AXB_C_Dm group had a CI value closest to 1, indicating better conformity. Additionally, the Gradient Measure (PTV_GM) was shorter in the AXB_C_Dw group, with a value of 2.995.For the D2%, D50%, and D98% dose differences within the PTV, significant differences were consistently observed between the AAA and AXB algorithms. Notably, the AAA algorithm yielded higher D50% and D98% values than the AXB algorithm under both enhanced and non-contrast enhanced CT.

**Figure 6:**
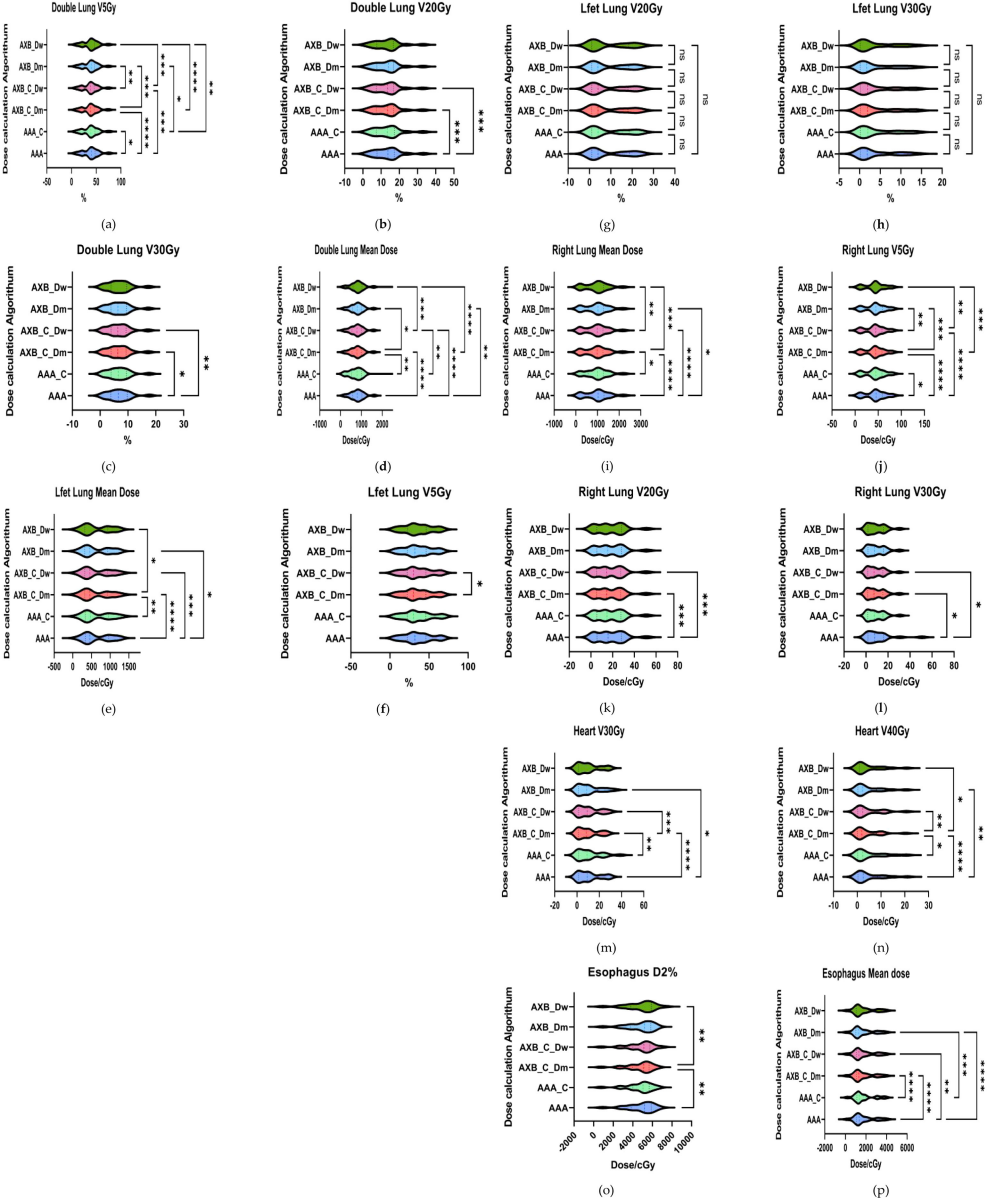
Results of evaluation parameters of OAR under different conditions. **(a–i)** respectively represent lung radiotherapy dose evaluation parameters, including the mean dose, V5, V20, and V30 **(m–n)** respectively represent cardiac radiotherapy dose evaluation parameters, including V30 and V40; **(o–p)** respectively represent esophageal radiotherapy dose evaluation parameters, including D2% and mean dose.where * indicates p<0.05, ** indicates p<0.01, *** indicates p<0.001, and **** indicates p<0.0001. The abbreviation “ns” stands for “not significant,” meaning p≥0.05.

The mean dose evaluation parameters and VX% values for various organs at risk (OARs) are summarized in **Figure 6**. Significant differences were observed in the dual-lung V5 dose between the AAA and AXB_C_Dm algorithms, as well as between the AXB_C_Dm and AXB_Dw algorithms (p< 0.0001). The mean dual-lung V5 value in the AAA group was higher than those in the AXB_C_Dm and AXB_Dw groups. Similarly, significant differences were found for dual-lung V20 and V30 doses across these algorithms (p< 0.05).For the left lung, significant differences were observed in the mean dose between the AAA and AXB groups (p< 0.0001), with the AAA algorithm yielding higher values. However, no statistically significant differences were found for the left lung V5, V20, or V30 doses. In the right lung, the mean dose and V5, V20, and V30 doses showed significant differences between the AAA and AXB algorithms (p< 0.05), with the AAA algorithm consistently predicting higher values. Notably, the contrast-enhanced CT condition slightly underestimated the irradiation dose ratio in the lung's low-dose region when using the AXB algorithm, particularly in the Dm and Dw dose reporting modes. In addition, we compared the dose distributions for two critical organs, the heart and esophagus, focusing on the maximum D2% and mean organ-at-risk (OAR) dose distributions for heart V30 and V40, as well as the esophagus. For heart V30 and V40, statistically significant differences (p< 0.05) were observed between the following groups: AAA vs. AXB_C_Dm, AAA vs. AXB_Dm, AAA_C vs. AXB_C_Dm, and AXB_C_Dm vs. AXB_C_Dw. The more pronounced differences were primarily attributed to variations between the different algorithmic models, particularly between the two dose-reporting modes of the AXB algorithm under the enhanced CT condition. Specifically, the V30 and V40 values in the AXB_C_Dw group were 10.44% and 4.685%, respectively, which were significantly higher than the corresponding values of 9.55% and 4.27% in the AXB_C_Dm group.

As shown in **Figure 6** (Esophagus D2%), comparisons of esophageal D2% revealed statistically significant differences (p< 0.05) between the AAA vs. AXB_C_Dm and AXB_C_Dm vs. AXB_Dw groups. Additionally, comparisons of the esophageal mean dose showed significant differences among the following groups: AAA vs. AXB_C_Dm, AAA vs. AXB_C_Dw, AAA vs. AXB_Dm, AAA_C vs. AXB_C_Dm, and AAA_C vs. AXB_Dm. These findings underscore that the choice of algorithm and dose-reporting mode can significantly influence dose distribution assessments for critical organs.

### 3.3 Analysis of tumor control probability

**Figure 7:**
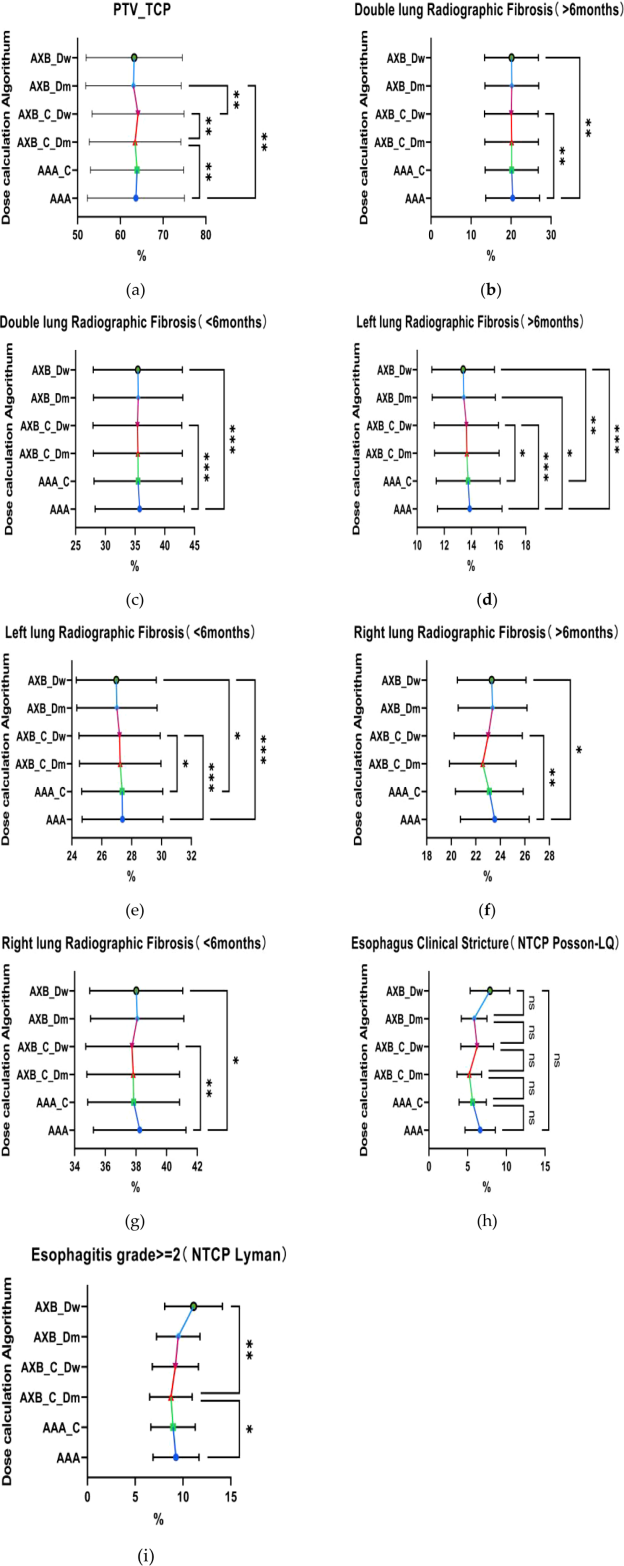
Comparison of tumor control probability (TCP) and normal tissue complication probability (NTCP). **(a)** Tumor Control Probability (TCP) of the target volume PTV, **(b–e)** respectively represent the Normal Tissue Complication Probability(NTCP) for both lungs and the left lung, indicating the probability of developing radiation-induced fibrosis within six months or after six months. **(h–i)** represent the comparison of the NTCP for the esophagus.where * indicates p<0.05, ** indicates p<0.01, *** indicates p<0.001. The abbreviation “ns” stands for “not significant,” meaning p≥0.05.

The result of TCP analysis demonstrates that the AAA algorithm consistently predicts higher TCP values compared to the AXB_Dm algorithm, a trend observed across both imaging modalities, as shown in **Figure 7**. This suggests that the AAA algorithm may have a tendency to overestimate tumor control relative to AXB_Dm, which could significantly impact clinical decisions regarding treatment efficacy. The observed statistically significant differences (p = 0.0025, 0.0093) highlight the substantial influence that the choice of dose calculation algorithm can have on TCP predictions. Further, the impact of imaging modalities, particularly under the contrast-enhanced CT (CECT) condition, reveals significant differences in TCP values between the two dose-reporting modes of the AXB algorithm (AXB_C_Dm and AXB_C_Dw) (p=0.0011). Specifically, the TCP values were found to be lower in the AXB_C_Dm group compared to the AXB_C_Dw group by 0.69%. Moreover, the presence of a contrast agent appeared to amplify these differences in dose calculation, as evidenced by the 1.08% lower TCP values in the AXB_Dm group under non-contrast enhanced CT compared to the AXB_C_Dw group. These findings underscore the critical role of both algorithm selection and imaging modality in accurately predicting TCP, which in turn, can guide more effective and individualized radiotherapy treatment planning.

### 3.4 The analysis of normal tissue complication probability

The analysis of NTCP revealed that the AAA algorithm predicted significantly higher NTCP values for radiofibrosis in both lungs compared to the AXB_C_Dw and AXB_Dw groups, as shown in the **Figure 7**, with the AAA group showing an NTCP value that was 20.42% higher, suggesting a greater predicted risk of radiation-induced lung complications. When focusing on the left lung, the temporal and spatial analysis further indicated that the AAA algorithm consistently predicted higher NTCP values for radiofibrosis over different time periods, whether greater or less than six months, with statistically significant differences (p=0.0007, 0.0003), where the NTCP values were 0.25% and 0.49% higher than those predicted by the AXB_C_Dw and AXB_Dw groups, respectively. Additionally, although there were no significant differences in the probability of developing clinical stricture in the esophagus across different algorithms or imaging modalities (p > 0.05), significant differences were observed in the probability of developing greater than grade 2 radiation esophagitis between the AAA and AXB_C_Dm groups, as well as between the AXB_C_Dm and AXB_Dw groups (p = 0.0126, 0.0018). These findings underscore the potential for the AAA algorithm to predict higher risks of radiation-induced complications, necessitating careful consideration in treatment planning to avoid excessive normal tissue damage.

## 4 Discussion

This study provides a critical analysis of the impact that different dose calculation algorithms (AAA and AXB) and imaging modalities (enhanced vs. non-contrast enhanced CT) have on radiotherapy dose distribution and radiobiological predictions, with a focus on lung cancer treatment. The findings underscore the significance of algorithm choice in clinical practice, particularly when using contrast-enhanced CT images, where discrepancies in dose calculations can lead to substantial variations in both TCP and NTCP.

The observed tendency of the AAA algorithm to overestimate doses, particularly in low-dose regions, is consistent with previous studies that have highlighted the algorithm's limitations in accurately accounting for tissue heterogeneities. For instance, Fogliata et al. demonstrated that the AAA algorithm often overestimates doses in regions where there are steep dose gradients, such as near lung-tissue interfaces, leading to potentially inaccurate treatment planning (27). This overestimation is particularly problematic in the presence of iodinated contrast agents, as reported by Smith et al, where AAA's inaccuracies are further amplified, potentially leading to an unnecessary increase in the dose delivered to healthy tissues and, consequently, a higher risk of radiation-induced toxicities (28). This is crucial, as contrast-enhanced imaging is commonly used to improve tumor visualization and delineation during planning. However, the high atomic number of iodine alters the attenuation properties of tissues, significantly affecting the photon energy spectrum. AAA, due to its empirical nature and lack of energy spectrum corrections for high-Z materials, struggles to adjust for these changes, leading to overestimation of doses in tissues where contrast agents accumulate. Smith et al.’s observations regarding AAA’s performance in the presence of contrast agents emphasize the need for algorithms that can accurately model the altered physics introduced by these agents (29–31). The AXB algorithm, particularly in its dose-to-water mode, has been shown to provide more accurate dose calculations under enhanced CT conditions. Krieger et al. found that AXB outperforms AAA in scenarios involving complex tissue compositions, such as those found in lung cancer patients, where accurate dose calculation is crucial for minimizing normal tissue complications (32–34). AXB directly solves the Linear Boltzmann Transport Equation (LBTE), making it particularly well-suited for dose calculation in heterogeneous tissues such as the lung. Multiple prior studies have shown that AXB closely approximates MC-calculated dose distributions. For example, Bush et al. demonstrated high agreement between AXB and MC in lung phantom models, with discrepancies typically within 2% (35). Similarly, Fogliata et al. and Vassiliev et al. reported that AXB outperforms convolution/superposition algorithms like AAA, particularly in low-density regions and air–tissue interfaces. These findings support our use of AXB as a surrogate benchmark in the current comparative analysis (27, 36).The current study’s findings, which indicate significantly lower NTCP values for lung complications using the AXB algorithm compared to AAA, align with this earlier research. The superior performance of AXB in predicting radiobiological outcomes, particularly in the context of enhanced CT imaging, suggests that it should be considered the preferred algorithm in clinical settings where precision is paramount.

The variability in TCP and NTCP predictions between AAA and AXB algorithms has significant clinical implications. The consistent overestimation of TCP by the AAA algorithm, particularly under CECT conditions, could lead clinicians to overestimate tumor control, potentially resulting in less aggressive treatment plans for tumors that might require more intensive intervention. Some Researchers highlighted that differences in algorithmic predictions could lead to significant variability in clinical outcomes, underscoring the need for critical evaluation of TCP values during treatment planning (35). Furthermore, the introduction of contrast agents appears to exacerbate the differences between these algorithms, particularly in low-dose regions. Such discrepancies could result in overestimation of normal tissue complications, such as radiation pneumonitis, when using the AAA algorithm (37, 38). This finding is echoed in our study, where the AAA algorithm predicted significantly higher NTCP values for lung fibrosis compared to the AXB_C_Dw group, suggesting that the use of AAA might necessitate more conservative treatment approaches to mitigate the risk of radiation-induced side effects. The lack of significant differences in the probability of developing clinical esophageal strictures across different algorithms may initially seem incongruent with the overall trend of AAA's overestimation. While AAA tends to overestimate doses in inhomogeneous tissue compositions, the clinical manifestation of complications like esophageal strictures may be influenced by other factors beyond dose calculation alone, such as the patient's baseline esophageal condition and the specific radiobiological characteristics of the esophagus. Although several comparisons of TCP and NTCP values between AAA and AXB showed statistically significant differences (p< 0.05), the majority of absolute differences were below 1%. From a clinical perspective, such small variations are unlikely to meaningfully influence treatment decisions, particularly when considering routine margins for organ-at-risk (OAR) tolerance and tumor control thresholds. Therefore, while the presence of iodinated contrast and algorithm choice can lead to measurable differences, only those exceeding clinically relevant thresholds (typically >3-5% for NTCP or >5% for TCP in critical cases) should be considered for influencing practice (39, 40). These findings underscore the importance of not only statistical testing but also clinical judgment when interpreting radiobiological predictions in treatment planning. While some of the observed differences in TCP and NTCP values were statistically significant, many were below 1%, which likely limits their clinical impact in standard radiotherapy workflows. However, larger differences—such as >20% variations in NTCP for lung fibrosis or >1–2% changes in TCP in dose-intensive regions—could influence clinical decision-making in select cases, especially when considering dose escalation, OAR constraints, or patients with compromised organ function. These findings underscore the need to interpret radiobiological metrics alongside comprehensive clinical evaluation, rather than in isolation.

It is important to acknowledge a methodological limitation of this study concerning the comparison of NTCP and TCP values derived from AAA- and AXB-based dose calculations. In this study, the treatment plan was optimized using the AAA algorithm, and the AXB dose distribution was obtained by recalculating the same plan without re-optimization. While this approach ensures consistency in beam geometry and monitor units, it may introduce a potential bias in the NTCP estimates, particularly because NTCP is sensitive to dose distributions within organs at risk (OARs).Since AXB was not independently optimized to minimize OAR doses, its NTCP values may reflect a more conservative or lower risk simply because the dose distribution is inherently different from that of AAA, without the benefit of optimization to further spare healthy tissue. As a result, comparisons of NTCP between the two algorithms should be interpreted with caution. However, our primary objective was to compare how the two dose calculation algorithms perform under identical planning conditions—thereby isolating the impact of the algorithm itself rather than the influence of planning strategies. This approach aligns with the clinical reality in many institutions, where a single optimization algorithm is used, and secondary algorithms serve as recalculation tools for quality assurance or validation. Future studies may benefit from including fully re-optimized AXB-based plans to enable a more direct comparison of TCP/NTCP values under algorithm-specific planning conditions.

It is crucial for future research to continue exploring the impact of different dose algorithms on a broader range of radiobiological effects, including long-term outcomes such as secondary malignancies and late-onset toxicities. Moreover, as advanced imaging techniques and novel dose algorithms continue to develop, ongoing validation studies are essential to ensure that treatment plans are both accurate and individualized, ultimately improving the therapeutic ratio in radiotherapy.

## 5 Conclusions

In the presence of contrast agents during lung radiotherapy, the AXB algorithm, especially in its dose-to-water reporting mode, is recommended for treatment planning. This algorithm provides more accurate dose calculations, avoids the overestimation of TCP and NTCP calculated with the AAA algorithm, and ensures a more precise balance between effective tumor treatment and minimizing risks to normal tissues. The use of the AXB algorithm under these conditions is crucial to optimize patient outcomes and reduce the likelihood of radiation-induced complications.

## Data Availability

The original contributions presented in the study are included in the article/supplementary material. Further inquiries can be directed to the corresponding author.
